# Nurses’ perspectives on implementing pediatric palliative care: a qualitative participatory study on the emerging role of the advanced practice nurse

**DOI:** 10.1007/s00431-026-06790-0

**Published:** 2026-02-21

**Authors:** Simone Saringer-Siegl, Roland Eßl-Maurer, Piret Paal

**Affiliations:** 1 Nursing Department, University Hospital Salzburg, Paracelsus Medical University, Salzburg, Austria; 2 Department of Pediatric, University Hospital Salzburg, Paracelsus Medical University, Salzburg, Austria; 3 Department of Ethnology, Institute of Cultural Research, Tartu University, Tartu, Estonia

**Keywords:** Pediatric Palliative Care, Nursing, Participatory research, Implementation, Support needs

## Abstract

The implementation of Pediatric Palliative Care (PPC) in hospital settings places considerable demands on nursing staff, particularly during early phases of change. This study explored nurses’ experiences, attitudes, and perceived support needs during the early implementation of a PPC concept at a tertiary pediatric hospital in Austria. A qualitative participatory design was applied. Four moderated focus group discussions were conducted with 18 registered nurses working on two pediatric wards where PPC beds were newly introduced. Data were audio-recorded, transcribed verbatim, and analyzed using Braun and Clarke’s thematic analysis. Four themes were identified: experiences with PPC, PPC as a new model of care, challenges of implementation, and expectations toward PPC. Nurses reported emotional burden, conceptual uncertainty, and limited opportunities for structured training and reflection. Although many participants had prior experience caring for children with life-limiting conditions, these experiences were largely informal and lacked a shared framework. Nurses articulated a strong need for accessible support structures and identified the absence of advanced nursing expertise as a key gap during early PPC implementation. *Conclusion*: Nurses’ experiences underscore the importance of structured support, conceptual clarity, and emotional resources during early PPC implementation. The articulated need for advanced nursing expertise emerged inductively as a support requirement rather than an evaluation of an existing role and may inform future implementation and role development strategies.

**Table:** 

**What is Known:** *• Pediatric Palliative Care is frequently misunderstood as synonymous with end-of-life care.* *• Implementing Pediatric Palliative Care in hospital settings requires complex organizational change.*
**What is New:** *• This qualitative study provides in-depth insight into nurses’ experiences during the early implementation of Pediatric Palliative Care in a tertiary pediatric hospital.* *• Although nurses had prior informal experience caring for children with life-limiting conditions, PPC was perceived as both familiar and novel due to the absence of a shared conceptual framework, leading to emotional burden, conceptual uncertainty, and unmet needs for structured support, including accessible advanced nursing expertise.*

**Supplementary Information:**

The online version contains supplementary material available at 10.1007/s00431-026-06790-0.

## Introduction

While childhood mortality rates in high-income countries have declined substantially, the prevalence of pediatric life-limiting conditions is increasing. This trend is partly attributable to advances in medical care, which allow children with complex and previously fatal conditions to survive into childhood and adolescence, often with ongoing palliative care needs [1, 2]. As a result, healthcare systems face growing emotional, organizational, and professional challenges.

Pediatric Palliative Care (PPC) is a holistic, multidisciplinary approach that aims to improve quality of life for children with life-limiting conditions and their families by addressing medical, psychological, social, and spiritual needs [3–6]. The World Health Organization emphasizes that PPC should be integrated early in the illness trajectory and embedded within routine pediatric care [5]. However, the implementation of PPC requires substantial cultural and organizational change within hospital settings and remains challenging in practice.

International research consistently highlights barriers to PPC implementation, including limited awareness, emotional resistance, insufficient resources, and gaps in professional education [6–11]. Studies from the United Kingdom, Switzerland, Norway, and Australia show that healthcare professionals often associate PPC with death and dying, leading to delayed referrals, uncertainty, and emotional strain [6–12]. Across settings, nurses emphasize the need for structured education, emotional support, and clearly accessible expertise during PPC implementation.

Despite this growing body of literature, empirical evidence from continental Europe remains limited. This gap is notable given the expansion of PPC programs across Europe and ongoing calls to examine implementation processes, interprofessional collaboration, and role development in diverse healthcare systems [12–17]. In Austria, national frameworks for PPC exist, yet little is known about how frontline nursing staff experience the introduction of PPC concepts in hospital practice.

While the role of Advanced Practice Nurses (APNs) in PPC has been discussed conceptually at an international level [13–15], empirical data on their relevance during implementation processes—particularly in German-speaking healthcare contexts—are scarce. Importantly, the present study did not aim to evaluate an implemented APN role. Rather, nurses’ perspectives on advanced nursing expertise emerged inductively from the data as a perceived gap and support need during the early phase of PPC implementation.

This study addresses this research gap by exploring nurses’ experiences, attitudes, and perceived support needs during the early implementation of PPC at a tertiary children’s hospital in Austria. By situating nurses’ perspectives within the context of international evidence, the study contributes context-specific insights into the challenges of PPC implementation in hospital settings.

The study aimed to explore nurses’ experiences, attitudes, and perceived support needs during the early implementation of PPC, with particular attention to how nurses described existing support structures and articulated needs for advanced nursing expertise [21, 22]. At the time of data collection, a fully established APN role within the PPC program had not yet been implemented.

## Materials and methods

### Study design

A qualitative participatory research design was used to explore nurses’ experiences during PPC implementation. The participatory approach was reflected across several stages of the study. Clinical nurses contributed to shaping the study focus through informal preliminary discussions that informed the development of the semi-structured interview guide. Focus group discussions were conducted in a dialogical manner, allowing participants to actively raise issues they perceived as relevant. During analysis and interpretation, emerging themes were discussed within the multidisciplinary research team, including members with clinical nursing backgrounds, to ensure findings were grounded in practice.

### Setting and participants

In 2022, the Children’s Center of the University Hospital Salzburg, a tertiary pediatric hospital in Austria, initiated the structured implementation of a PPC concept, including the introduction of dedicated PPC beds on two pediatric wards (a Neonatal and Infant Ward and a Parent–Child Ward). In parallel, a multiprofessional PPC team was established, comprising professionals from medicine, nursing, pastoral care, physiotherapy, speech and language therapy, psychology, social work, and dietetics.

At the time of data collection, the PPC team was formally constituted but still in an early developmental phase. Standardized workflows, referral pathways, and routine collaboration patterns with ward-based teams had not yet been fully established. The team was intended to function in a consultative and supportive capacity, while primary responsibility for daily palliative care remained with ward-based nursing and medical staff. As a result, interactions between the PPC team and ward nurses varied and evolved on a case-by-case basis.

Purposive sampling was used to recruit registered nurses working on the two wards where PPC beds were implemented. Eligible participants had at least six months of professional experience on the respective ward and were directly involved in the care of children with life-limiting or complex chronic conditions. Nurses were excluded if they were not involved in inpatient care, were on extended leave during the data collection period, or had no clinical contact with the target patient group.

A total of 18 nurses (predominantly female, aged 24–55 years) participated. All nurses who consented completed the study, and no withdrawals or missing data occurred. Participants were informed about the study through verbal and written information on the wards. The first author introduced the study during team meetings and through direct contact, emphasizing voluntary participation and study procedures. Written informed consent was obtained from all participants prior to data collection.

### Data collection

Nurses were recruited using purposive sampling from the two pediatric wards where PPC-beds were implemented. The study was introduced by the first author during ward-based team meetings and through direct personal contact. Nurses received verbal and written information about the study aims, procedures, and voluntary nature of participation, and written informed consent was obtained prior to data collection.

Data were collected through four moderated focus group discussions (4–5 participants each) conducted in November 2022. The focus groups were moderated by a pediatric nurse with advanced nursing training who was familiar with the clinical setting but did not hold a supervisory role over participants. This relationship was acknowledged and addressed through reflexive awareness to minimize potential influence on group dynamics.

Discussions were held in a private meeting room at the hospital, lasted 60–90 min, and followed a semi-structured interview guide informed by existing literature and clinical insights [1–10]. The guide explored prior experiences with PPC, perceptions of the new care model, challenges during implementation, and perceived support needs. All discussions were audio-recorded and transcribed verbatim.

### Data analysis

Focus group discussions were chosen to facilitate interaction and collective reflection among nurses working in similar clinical contexts, enabling participants to build on shared experiences and discuss emotionally sensitive topics related to PPC [24, 25].

Data were analyzed using Braun and Clarke’s six-phase thematic analysis [26]. The first author conducted initial coding and theme development, supported by peer debriefing with the co-authors to enhance analytic rigor. Coding decisions and theme development were documented in an audit trail, and discrepancies were resolved through consensus. MAXQDA 2022 software was used to organize and manage the data.

Thematic saturation was considered achieved when no new themes or subthemes emerged across successive focus groups. Initial discussions generated the core thematic structure, while subsequent groups served to confirm and refine existing themes. The inclusion of nurses with varying professional experience from both wards supported thematic saturation within the scope of the study.

### Reflexivity

The research team comprised professionals with backgrounds in pediatric nursing, advanced nursing practice, and ethics. The first author held a dual role as clinician and researcher within the study setting, facilitating trust and access while requiring ongoing reflexivity to address potential bias.

Reflexive strategies included peer debriefing, collaborative coding, and regular critical discussions within the multidisciplinary research team to ensure that interpretations were grounded in participants’ accounts rather than researchers’ assumptions.

### Ethical considerations

Ethical approval was obtained from the Ethics Committee of the Paracelsus Medical University (SS22-0046–0046). Written informed consent was secured from all participants.

## Results

The focus group data were analyzed using Braun and Clarke’s six-phase thematic analysis [26]. This approach enabled a systematic identification of patterns across the dataset. Four main themes and 13 subthemes were identified (Table 1), reflecting nurses’ experiences, perceptions, and expectations regarding PPC implementation. Selected illustrative quotes are presented below; additional quotes and extended descriptions are provided in the Online Supplementary Materials.

**Table 1:** Overview of themes and subthemes

Theme	Subthemes
1. Experiences with PPC	1a) Longstanding presence of PPC practices
	1b) Lack of training
	1c) Avoiding the taboo of death
	1 d) Need for structured support and training
2. PPC as a New Model of Care	2a) Conceptual confusion
	2b) 'Palliative' as a trigger for fear
	2c) Recognition of PPC as life-long care
3. Challenges of Implementation	3a) Emotional burden
	3b) Managing parental expertise and expectations
	3c) Time limitations
4. Expectations Toward PPC	4a) Communication strategies
	4b) Role and expectations of the PPC team
	4c) Desired support through an APN

In the following sections, each theme is presented with illustrative participant quotes and interpretive commentary to convey both the content and the emotional nuances of the findings.

### Theme 1: Experiences with Pediatric Palliative Care

Nurses reported longstanding experience in caring for children with complex and life-limiting conditions, even before the formal implementation of PPC. Many participants emphasized that palliative situations had always been part of their daily practice, although they were not explicitly labeled or structured as PPC.

“I’ve been working with patients like this for a long time. At the beginning, I often thought they were completely alone, with no support at all.” (Group II)

#### 1a) Longstanding presence of palliative practices

Several nurses described that core elements of palliative care—such as symptom management, family support, and emotional accompaniment—had long been integrated into routine care. However, these practices were largely implicit and lacked a shared conceptual or organizational framework.

#### 1b) Lack of training

Despite extensive practical experience, many participants reported limited formal education in PPC. Differences emerged depending on prior exposure: nurses with long-standing experience caring for children with complex conditions perceived PPC as partly familiar, whereas less experienced nurses expressed greater uncertainty and emphasized the absence of structured training.

#### 1c) Avoiding the taboo of death

Open discussions about death and dying were described as challenging within teams and in communication with families. This avoidance contributed to uncertainty and discomfort, particularly in emotionally demanding situations.

####  1d) Need for structured support and training

Beyond education, nurses emphasized the need for structured support, including opportunities for reflection, team discussions, and emotional processing when dealing with severe illness and end-of-life issues.

#### Integrated thematic summary

Although many nurses had longstanding experience caring for children with life-limiting conditions, these experiences were largely unstructured and not embedded within a shared PPC framework. Limited formal training, avoidance of death-related conversations, and a lack of structured support contributed to emotional insecurity during early PPC implementation.

### Theme 2: Pediatric Palliative Care as a new model of care

Although PPC was generally viewed as valuable, nurses described it as an unfamiliar and emotionally charged model of care during early implementation. Participants reported uncertainty regarding the scope, timing, and practical implications of PPC within routine pediatric care.

#### 2a) Conceptual confusion

Many nurses expressed difficulty in clearly defining what PPC encompassed and how it differed from existing care practices. Uncertainty existed regarding professional responsibilities and the appropriate timing of PPC involvement, particularly in non-terminal situations.

#### 2b) “Palliative” as a trigger for fear

The term “palliative” was frequently associated with death and dying, which generated discomfort and resistance among staff and families. This association reflected longstanding institutional practices in which death was perceived as something to be avoided within pediatric wards.

“Death was not permitted to occur on our ward either… patients would be transferred elsewhere beforehand.” (Group IV)

#### 2c) Recognition of PPC as life-long care

Over time, some participants began to reconceptualize PPC as a long-term, family-centered approach rather than care limited to the end of life. This shift in understanding was described as a gradual process influenced by clinical experience and exposure during implementation.

#### Integrated thematic summary

This theme captures nurses’ process of reframing PPC from a death-centered concept toward a life-supporting model of care. Conceptual uncertainty and emotionally loaded language initially shaped resistance, while early implementation experiences contributed to a more nuanced understanding of PPC as an integral component of pediatric care.

### Theme 3: Challenges of implementation

The implementation of PPC was described as emotionally, communicatively, and organizationally demanding. Nurses reported that integrating palliative principles into routine hospital care intensified emotional strain and exposed structural limitations during the early implementation phase.

#### 3a) Emotional burden

Participants consistently described emotional exhaustion, insecurity, and fear of providing inadequate care, particularly when accompanying children and families through prolonged illness trajectories and situations of prognostic uncertainty.

“I feel highly insecure and fear that I am providing suboptimal care. I try to avoid this type of care as much as possible.” (Group IV)

These experiences sometimes led to avoidance behaviors and reinforced feelings of inadequacy.

#### 3b) Managing parental expertise and expectations

Interactions with well-informed and emotionally involved parents were perceived as particularly challenging. While parental knowledge and advocacy were valued, nurses reported increased pressure to meet high expectations regarding clinical accuracy, communication, and emotional availability—especially in complex decision-making situations.

“You feel under pressure because parents know so much and question every step.” (Group II)

The challenge was not parental expertise itself, but the lack of clear roles, communication structures, and accessible support during early PPC implementation.

#### 3c) Time limitations

Time constraints were described as a major barrier to effective PPC delivery. High workload and competing priorities limited nurses’ ability to engage in meaningful conversations and provide emotional presence.

“You know these families need more time, but in reality, you just don’t have it.” (Group I)

#### Integrated thematic summary

This theme highlights how emotional burden, challenging interactions with highly involved parents, and persistent time constraints shaped nurses’ experiences during early PPC implementation. These challenges underscore the need for institutional structures that address both emotional support and organizational resources.

### Theme 4: Expectations toward Pediatric Palliative Care

Looking ahead, nurses articulated clear expectations regarding how PPC should be supported and structured within the institution. Participants emphasized the need for improved communication, visible team support, and accessible expertise to navigate the emotional and clinical demands of PPC.

#### 4a) Communication strategies

Nurses highlighted the importance of regular, structured communication within teams and opportunities for brief reflection in daily practice. Even short, informal exchanges were perceived as valuable for sharing concerns and maintaining emotional well-being.

“Communication is key. Even five minutes during lunch to check in would help.” (Group I).

#### 4b) Role and expectations of the PPC team

Participants expected the PPC team to be visible, approachable, and actively involved in care planning and team support. Nurses emphasized the importance of having a clearly identifiable contact point for guidance in complex clinical and ethical situations.

#### 4c) Desired support through advanced nursing expertise

Importantly, participants were not reflecting on experiences with an established Advanced Practice Nurse (APN) role. Rather, they described the perceived absence of accessible advanced nursing expertise during early PPC implementation. Nurses expressed a general desire for reliable support when facing complex decisions, emotionally demanding situations, and uncertainty.

“Sometimes you feel left alone with very difficult decisions and emotions.” (Group II)

At the time of data collection, nurses were unable to clearly define specific APN role functions, as advanced nursing roles were still under development and not yet embedded in practice. The APN was therefore envisioned as a currently missing source of support rather than an existing or insufficiently developed role.

#### Integrated thematic summary

This theme captures nurses’ expectations for supportive structures during PPC implementation, including effective communication, visible team support, and the future integration of advanced nursing expertise to address unmet clinical and emotional needs.

Additional illustrative quotes and extended descriptions for the themes are provided in Online Supplementary Material.

## Discussion

### Early implementation context and nurses’ experiences

This study illustrates the complexity of implementing PPC within a tertiary pediatric hospital setting. Nurses described substantial emotional burden, conceptual uncertainty, and a pronounced need for structured guidance during the early phase of implementation. Although a multiprofessional PPC team was established, primary responsibility for daily palliative care remained with ward-based nursing staff, contributing to increased responsibility and emotional strain.

Many nurses reported longstanding experience caring for children with life-limiting conditions prior to the formal introduction of PPC. However, these experiences were largely informal and lacked a shared conceptual or organizational framework. As reflected in the Results, palliative situations had often been part of everyday practice “without a name,” helping to explain why PPC was perceived as both familiar and novel during implementation.

At the time of data collection, the PPC team itself was still in an early developmental phase, with roles and collaboration pathways not yet fully defined. The absence of established routines and clear points of contact contributed to nurses’ uncertainty and reinforced their perception of increased responsibility.

### Involving and supporting nurses during implementation

A key finding was the distinction between lack of training and the need for structured support. While training addresses knowledge and skills, structured support refers to organizational and relational frameworks that enable reflection, shared responsibility, and the processing of emotional and moral distress. Nurses’ accounts suggest that education alone is insufficient without parallel structures that provide emotional containment during PPC implementation.

These findings align with international literature documenting emotional burden, compassion fatigue, and moral distress among pediatric nurses in palliative settings [7, 10, 16–18, 20, 23, 29, 30]. Differences related to prior experience also emerged: nurses with longstanding exposure to complex care described greater familiarity with PPC principles, whereas less experienced nurses expressed stronger uncertainty and emphasized the need for structured training—echoing international findings [7, 10, 12].

### Pediatric Palliative Care as a model of care

Consistent with studies from the UK, Switzerland, Norway, and Australia [10, 16–20, 28], PPC was initially associated with end-of-life care, contributing to conceptual uncertainty and attitudinal barriers. These findings underscore the importance of targeted education and cultural reframing to support the early integration of PPC as a longitudinal, family-centered model of care.

### Emergent need for advanced nursing expertise

During early implementation, nurses were expected to integrate palliative principles more explicitly into routine care, including earlier identification of needs and intensified family communication. While the PPC team offered support, many nurses experienced increased responsibility, highlighting the need for accessible, ward-based expertise.

Importantly, the APN role was not evaluated as an implemented component of the PPC program. Nurses’ difficulty in articulating concrete role functions reflects the early timing of data collection, which coincided with the initial implementation of PPC beds and parallel role development. Consequently, expectations toward advanced nursing expertise emerged inductively as a perceived support need rather than a predefined intervention.

Although international frameworks such as GO-PPaCS and the PEPPA model highlight the potential value of advanced nursing roles [5, 13–15], empirical evidence in PPC remains limited. In this study, nurses articulated expectations—such as clinical guidance, emotional support, and educational mentoring—that align with internationally described APN competencies [6, 9, 13]. At the same time, potential barriers, including limited resources, unclear role definitions, and restricted availability of advanced training, must be considered, particularly in German-speaking contexts.

### Implications for future research and measurment

Beyond structural and role-related aspects, the findings emphasize the importance of communication and relational competence in PPC [27]. Emerging measurement tools, such as the Visual CARE Measure [31] and the PACPN instrument [32], may support future mixed-method research by capturing perceptions of empathy and emotional support.

Overall, this study contributes empirical insights from Austria that corroborate known barriers while highlighting nurses’ perceptions of unmet support needs during early PPC implementation. Rather than evaluating an established intervention, the findings point to advanced nursing expertise as a potential future strategy articulated by nurses to support sustainable integration of pediatric palliative care in hospital settings.

## Strengths and limitations

A key strength of this study lies in the rigor of its qualitative analysis. Coding and theme development were conducted systematically using qualitative analysis software (MAXQDA 2022), supported by an audit trail, peer debriefing, and consensus discussions within the research team. The absence of participant dropouts and missing data further strengthens the credibility of the findings.

Nurses’ prior informal exposure to palliative situations may have influenced how PPC was perceived; however, the lack of a shared framework before implementation underscores the relevance of examining experiences during this transitional phase. As data were collected during the early stage of implementation, conclusions about how nurses’ experiences may evolve once the PPC model or an APN role is fully established cannot be drawn. Longitudinal research is needed to explore changes over time.

Although this single-site study reflects a specific institutional context, the identified challenges align with international findings and may be transferable to similar pediatric hospital settings during early PPC implementation.

## Conclusion

Implementing PPC requires not only organizational change but also emotional readiness, cultural reframing, and sustained institutional support. This study provides empirical insights from an Austrian tertiary pediatric hospital, highlighting nurses’ emotional burden, conceptual uncertainty, and need for structured guidance during early PPC implementation.

Rather than evaluating an established role, the findings indicate that nurses articulated a perceived need for advanced nursing expertise to support clinical practice, communication, and emotional coping during this transitional phase. Future research should examine how such support structures, including advanced nursing roles, influence nurses’ experiences once PPC models are fully established and embedded in different healthcare contexts.

## Supplementary Information

Below is the link to the electronic supplementary material.

Supplementary file1 (DOCX 22 KB)

## Data Availability

The data is password-protected and stored in a neutral location. It is not possible for anyone to draw conclusions about a person based on the written group discussion. All anonymized data of the group discussions and their transcription as well as the declarations of consent are secured and stored by the researcher against unauthorized access.

## Abbreviations

APN: Advanced Practice Nurse
GO-PPaCS: Global Overview—Pediatric Palliative Care Standards
PEPPA: Participatory, Evidence-based, Patient-focused Process for the APN Role Development, Implementation, and Evaluation
PPC: Pediatric Palliative Care
WHO: World Health Organization
TA: Thematic Analysis
