# Supplementary material for: Nurses’ perspectives on implementing pediatric palliative care: a qualitative participatory study on the emerging role of the advanced practice nurse
Source: Eur J Pediatr. 2026 Feb 21;185(3):143. doi: 10.1007/s00431-026-06790-0 (PMC12924784; doi:10.1007/s00431-026-06790-0)
Supplement: Supplementary file 1 — Supplementary file1 (DOCX 22 KB) [file 431_2026_6790_MOESM1_ESM.docx]

**Online Supplementary Material**

**Expanded qualitative findings and illustrative quotes**

**Theme 1: Experiences with Pediatric Palliative Care**

This supplementary section provides additional illustrative quotes and extended descriptions supporting **Theme 1** as presented in the main manuscript. The material offers deeper insight into nurses’ experiences with pediatric palliative care prior to and during early implementation, without introducing new themes or interpretations.

**1a) Longstanding presence of palliative practices**

Many participants described that caring for children with life-limiting and complex chronic conditions had long been part of their everyday work, even before the formal introduction of pediatric palliative care. Several nurses emphasized that such children had “always been there,” although their care had not been explicitly conceptualized as palliative.

*“These children have always been on our ward. We just never called it palliative care.”* (Group I)

*“You did what you thought was right for the child and the family, but there was no common understanding or structure behind it.”* (Group III)

Nurses described providing symptom relief, emotional support, and close family involvement as routine aspects of care. However, these practices were largely shaped by individual experience rather than shared standards or institutional guidance.

**1b) Lack of training**

Despite extensive hands-on experience, many participants reported a lack of formal education in pediatric palliative care. Several nurses expressed uncertainty about whether their approach aligned with palliative principles and reported learning “by doing” rather than through structured training.

*“I never had any proper training in palliative care. You just grow into it somehow.”* (Group II)

Differences emerged depending on professional experience. Nurses with long-standing exposure to children with complex conditions felt more confident in managing palliative situations, whereas less experienced nurses expressed greater insecurity and a stronger desire for structured education.

*“For those who are newer, it’s much harder. You don’t know what’s expected of you.”* (Group IV)

**1c) Avoiding the taboo of death**

Participants consistently described death and dying as sensitive and often avoided topics within the clinical setting. This avoidance was present both in team communication and in interactions with families.

*“Death was something you didn’t really talk about openly on the ward.”* (Group I)

Several nurses noted that the lack of open dialogue contributed to uncertainty and discomfort, particularly when children’s conditions deteriorated or when families raised end-of-life questions.

*“You feel that it’s there, but no one really names it.”* (Group III)

**1d) Need for structured support and training**

Beyond formal education, nurses emphasized the need for structured support mechanisms to cope with emotionally demanding situations. Participants described a lack of regular opportunities for reflection, debriefing, or shared decision-making within the team.

“Sometimes you just need a space to talk about what you’ve experienced.” (Group II)

“It’s not only about knowledge. It’s about having support when things become emotionally overwhelming.” (Group IV)

Nurses expressed that such structures could help normalize emotional responses, reduce feelings of isolation, and foster a shared understanding of palliative care within the team.

**Supplementary summary**

Overall, the supplementary findings reinforce that nurses’ prior experiences with pediatric palliative situations were extensive but largely unstructured. The absence of formal training, open communication about death, and structured emotional support contributed to uncertainty and emotional strain, particularly during the early phase of PPC implementation. These detailed accounts complement the condensed presentation of Theme 1 in the main manuscript.

**
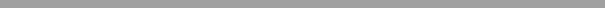
**

**Theme 2: Pediatric Palliative Care as a New Model of Care**

This supplementary section provides extended descriptions and additional illustrative quotes supporting Theme 2 as presented in the main manuscript. The material deepens understanding of nurses’ perceptions of pediatric palliative care (PPC) as a new and emotionally charged model of care during early implementation.

**2a) Conceptual confusion**

Participants frequently described uncertainty about what constituted pediatric palliative care and how it differed from established pediatric nursing practices. Several nurses reported difficulty identifying when PPC should be initiated and how it related to curative treatment approaches.

*“I wasn’t sure anymore where palliative care actually starts and where our normal care ends.”* (Group I)

Some nurses expressed concern that PPC lacked clear boundaries, which contributed to insecurity regarding their professional role and responsibilities during early implementation.

*“You suddenly ask yourself: Is this already palliative, or are we still just doing what we’ve always done?”* (Group III)

**2b) ‘Palliative’ as a trigger for fear**

The term “palliative” was repeatedly described as emotionally charged and closely associated with death and dying. Participants noted that this association often triggered unease among healthcare professionals and families alike.

*“As soon as you say ‘palliative,’ everyone thinks the child is going to die immediately.”* (Group II)

Several nurses described longstanding institutional practices in which dying children were transferred away from the ward, reinforcing the perception that death did not belong in routine pediatric care.

*“Death was not something that was supposed to happen on our ward.”* (Group IV)

This cultural context contributed to resistance toward early PPC integration and complicated communication with families.

**2c) Recognition of PPC as life-long care**

Through ongoing exposure during implementation, some nurses began to reframe PPC as a long-term, supportive approach rather than care limited to the terminal phase. This shift was described as gradual and closely linked to clinical encounters with children living for extended periods with complex conditions.

*“Only over time did I realize that palliative care doesn’t mean the end, but support over a long period.”* (Group I)

Participants described this reframing as an important learning process that reduced fear and supported more proactive engagement with families.

**Supplementary summary**

The supplementary findings illustrate how conceptual uncertainty and emotionally loaded language shaped nurses’ initial perceptions of pediatric palliative care. Over time, early implementation experiences supported a gradual reframing of PPC as a life-long, family-centered model of care. These extended accounts complement the condensed presentation of Theme 2 in the main manuscript.


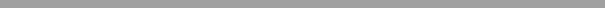


**Theme 3: Challenges of Implementation**

This supplementary section provides extended descriptions and additional illustrative quotes supporting Theme 3 as presented in the main manuscript. The material offers deeper insight into the emotional, communicative, and organizational challenges nurses experienced during the early implementation of pediatric palliative care (PPC).

**3a) Emotional burden**

Participants frequently described the emotional demands associated with caring for children with life-limiting conditions within the context of PPC implementation. Feelings of insecurity, helplessness, and fear of causing harm were particularly pronounced in situations characterized by prognostic uncertainty and ethical complexity.

*“You constantly wonder whether you’re doing the right thing for the child and the family.”* (Group III)

Several nurses reported that the emotional burden extended beyond end-of-life situations and accompanied long illness trajectories, contributing to cumulative emotional strain.

*“It’s not just one moment. It’s weeks or months of emotional tension.”* (Group I)

Some participants described avoidance behaviors as a coping strategy, reflecting a lack of emotional containment and support.

**3b) Managing parental expertise and expectations**

Interactions with parents were described as especially demanding when families arrived well-informed and highly emotionally involved in their child’s care. Nurses emphasized that parental expertise was generally respected and valued; however, the combination of high expectations, emotional intensity, and uncertainty created additional pressure.

*“Parents come with a lot of knowledge and emotions, and you feel you have to be perfect all the time.”* (Group II)

This pressure was intensified when parents actively questioned clinical decisions or compared information obtained from external sources with ward-based practices.

*“Sometimes parents know guidelines or studies, and you feel like you’re being examined.”* (Group IV)

Participants explained that managing parental expertise became particularly challenging in the absence of clear communication pathways, defined responsibilities, and accessible expert support during early PPC implementation.

**3c) Time limitations**

Time constraints were repeatedly identified as a structural barrier to providing high-quality PPC. Nurses described competing clinical demands, staffing shortages, and time pressure as limiting their ability to engage in in-depth conversations and provide emotional presence.

*“You know exactly what would be needed, but there’s simply no time for it.”* (Group I)

Several participants expressed frustration and moral distress when they were unable to meet their own professional standards due to time limitations.

*“That’s what makes it so exhausting—you want to do more, but the system doesn’t allow it.”* (Group III)

**Supplementary summary**

The supplementary findings reinforce that emotional burden, demanding interactions with highly involved parents, and persistent time constraints substantially shaped nurses’ experiences during early PPC implementation. These challenges highlight the importance of organizational structures that support emotional resilience, clarify roles, and allocate sufficient time for palliative care within pediatric hospital settings.


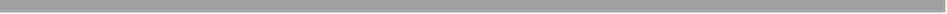


**Theme 4: Expectations Toward Pediatric Palliative Care**

This supplementary section provides extended descriptions and additional illustrative quotes supporting Theme 4 as presented in the main manuscript. The material elaborates on nurses’ expectations regarding supportive structures, communication, and advanced nursing expertise during early pediatric palliative care (PPC) implementation.

**4a) Communication strategies**

Participants repeatedly emphasized the importance of regular communication within and across teams. Nurses described a need for both formal and informal opportunities to exchange information, clarify uncertainties, and reflect on emotionally challenging situations.

*“Sometimes you just need a few minutes to talk things through with colleagues.”* (Group III)

Even brief moments of structured communication were perceived as helpful in reducing emotional burden and fostering a sense of shared responsibility.

*“It doesn’t always have to be a big meeting—sometimes five minutes are enough.”* (Group I)

**4b) Role and expectations of the PPC team**

Nurses expected the PPC team to be visible and approachable within everyday clinical practice. Participants described a desire for clear points of contact who could provide guidance in complex situations and support decision-making processes.

*“It would help to know exactly who to call when things become difficult.”* (Group II)

Several nurses expressed that uncertainty about how and when to involve the PPC team limited its perceived usefulness during early implementation.

*“You know there is a team, but you’re not always sure how to involve them.”* (Group IV)

**4c) Desired support through advanced nursing expertise**

Importantly, participants were not reflecting on experiences with an established Advanced Practice Nurse (APN) role. Rather, they articulated a perceived absence of accessible advanced nursing expertise during early PPC implementation. Nurses reported difficulty defining concrete role functions, as advanced nursing roles were still under development and not yet embedded in clinical practice.

*“I can’t really say what an APN would do exactly—but I know we would need someone like that.”* (Group I)

Instead of specific role descriptions, participants emphasized a general need for reliable support in challenging situations, including guidance during complex clinical decisions, assistance with difficult conversations, and emotional reassurance.

*“Sometimes you feel left alone with very difficult decisions and emotions.”* (Group II)

The APN was therefore envisioned as a future source of advanced nursing support rather than an existing or insufficiently developed role.

**Supplementary summary**

The supplementary findings illustrate nurses’ expectations for supportive structures during early PPC implementation. Participants emphasized the importance of communication, visible team support, and the future integration of advanced nursing expertise to address clinical uncertainty and emotional strain. These extended accounts complement the condensed presentation of Theme 4 in the main manuscript.
